# Target Trial Emulation to Incorporate Real-World Data in the Estimation of the Clinical and Cost-Effectiveness of Biologic Treatment

**DOI:** 10.1177/0272989X251408484

**Published:** 2026-01-15

**Authors:** Janharpreet Singh, Matt Stevenson, Kimme L. Hyrich, Clare L. Gillies, Keith R. Abrams, Sylwia Bujkiewicz

**Affiliations:** Biostatistics Research Group, Department of Population Health Sciences, University of Leicester, Leicester, UK; School of Health and Related Research, University of Sheffield, Sheffield, UK; NIHR Manchester Biomedical Research Centre, Manchester University NHS Trust, Manchester, UK; Centre for Epidemiology versus Arthritis, Manchester Academic Health Sciences Centre, The University of Manchester, Manchester, UK; Leicester Real World Evidence Unit, Leicester Diabetes Centre, Leicester General Hospital, University of Leicester, Leicester, UK; Department of Statistics, University of Warwick, Coventry, UK; Biostatistics Research Group, Department of Population Health Sciences, University of Leicester, Leicester, UK

**Keywords:** health technology assessment, real-world data, rheumatoid arthritis, target trial emulation

## Abstract

**Introduction:**

In the health technology assessment (HTA) of biologic treatments for rheumatoid arthritis (RA), there is limited randomized evidence on treatment effectiveness after first-line treatment failure. We demonstrate how real-world data (RWD) could fill this evidence gap.

**Methods:**

Target trial emulation (TTE) minimizes biases in the causal analysis of RWD by prespecifying a protocol for a hypothetical randomized clinical trial (RCT) that would estimate the effect of interest. The application of TTE for HTA was illustrated using RWD from the British Society for Rheumatology Biologics Register for Rheumatoid Arthritis to estimate the effectiveness of rituximab versus nonbiologic therapy (NBT) after first-line biologic failure, in terms of European Alliance of Associations for Rheumatology response achievement. The effectiveness estimates from RWD were combined with RCT estimates in a meta-analysis. The pooled estimates were entered into an economic model to estimate the incremental cost-effectiveness ratio (ICER) comparing biologic versus NBT strategies.

**Results:**

Based on RWD, rituximab was associated with higher probabilities of achieving a moderate or good response (0.215 v. 0.174) and a good response (0.090 v. 0.066) as compared with NBT. These probabilities were lower than those estimated from RCT data (moderate or good 0.650; good 0.150). The economic model estimated less time on treatment and lower costs associated with biologics when based on RWD compared with RCT data (mean £63,500 v. £70,000). This resulted in a higher ICER based on RWD compared with RCT data (mean £46,800 v. £34,700 per quality-adjusted life-year gained).

**Conclusions:**

RWD can provide supplemental evidence on treatment effectiveness where randomized evidence is limited. This can make a meaningful difference to cost-effectiveness estimates. Our results are not intended to inform current RA management.

**Highlights:**

- In health technology assessment, real-world data (RWD) can provide supplemental evidence on treatment effectiveness where there is limited randomized evidence.
- Target trial emulation was applied using RWD to estimate the clinical effectiveness of biologic treatment; these estimates were combined with estimates from an RCT in a meta-analysis, and the pooled estimates were entered into an economic model for rheumatoid arthritis.
- Treatment effect estimates based on combining RWD and RCT data were more modest compared with the effectiveness estimates from the RCT data alone, leading to a difference in the estimate of cost-effectiveness comparing biologics with nonbiologic therapy.

In health technology assessment (HTA), a comparison of the cost-effectiveness of multiple treatments requires modeling the lifetime costs and benefits associated with each treatment. For chronic conditions, such as rheumatoid arthritis (RA), this involves modeling an entire clinical pathway as individuals may switch through multiple lines of treatment due to a lack of a response (or an adverse event). Commonly, data are available from randomized controlled trials (RCTs) assessing the effectiveness of a first-line treatment, but data on the effectiveness of a treatment given after the first-line treatment may be more limited. In such cases, applying the cost-effectiveness analysis would require assumptions regarding the generalizability of the first-line treatment effect to subsequent lines of treatment or the use of nonrandomized evidence sources.^1^

The National Institute for Health and Care Excellence (NICE), in England and Wales, considers RCT data as the most reliable evidence on treatment efficacy (i.e., how well a treatment could work under optimal conditions) because random treatment allocation minimizes the risk of selection bias and bias due to confounding.^2^ However, such evidence can be limited for certain populations and is often not representative of treatment effectiveness (i.e., how well a treatment actually works in clinical practice), which is the key interest in an HTA.^3^ Real-world data (RWD), such as routinely-collected data from clinical practice (e.g., hospital appointments), recorded in a register could provide supplemental evidence on treatment effectiveness when analyzed appropriately.^4^ In particular, such studies collect data from a broader patient population, including individuals who progress through multiple lines of biologic treatment for RA.^5^

RWD are collected from nonrandomized sources and are at a higher risk of bias because individuals are allocated a treatment based on characteristics that may also be associated with the outcome used to measure the treatment effect. Consequently, estimating a treatment effect using RWD requires the application of statistical techniques for causal inference. Target trial emulation (TTE) involves defining a protocol for a hypothetical randomized trial to estimate a treatment effect based on the data that are available from the RWD source and applying this protocol when analyzing the RWD.^6,7^ Statistical techniques, such as inverse probability of treatment weighting (IPTW) based on propensity scores, can be applied to adjust for differences in measured characteristics between the treatment groups to mitigate confounding in the treatment effect.^8^ The adjusted treatment effect estimate should be more consistent with the causal effect that would have been observed in a randomized trial.

Before 2016, NICE clinical guidelines for RA recommended a first line of biologic treatment with 1 of the following disease-modifying antirheumatic drugs (DMARDs): an anti–tumor necrosis factor (TNF) drug, tocilizumab (TCZ), or abatacept (ABT). The second line of biologic treatment in this clinical pathway was rituximab (RTX). In an HTA, Stevenson et al.^9^ developed an economic model to compare the cost-effectiveness of the clinical pathway associated with the different first-line biologics based on these guidelines. This included modeling the costs and benefits for individuals taking RTX as a second line of biologic treatment in each pathway. In the model, the effectiveness of RTX as a second-line biologic was assumed equal to the effectiveness of ABT as a first-line biologic (see page 248 in Stevenson et al.^9^). This assumption was made based on the results of an indirect comparison from a previous HTA that found no significant difference in efficacy between RTX and ABT as second-line biologics for RA.^10^ In addition, a statistical mapping based on US register data was applied to obtain effectiveness estimates in terms of the European Alliance of Associations for Rheumatology (EULAR) response categories, which are used in UK clinical practice, from the American College of Rheumatology response criteria commonly reported in RCTs (see page 244 in Stevenson et al.^9^).

In this article, we aim to illustrate the TTE method using RWD from the British Society for Rheumatology Biologics Register for Rheumatoid Arthritis (BSRBR-RA)^11^ to estimate the effectiveness of RTX as a second line of biologic treatment for RA in terms of the EULAR response categories directly. We then enter the effect estimates based on RWD, those based on RCT data alone, and RWD + RCT data combined into an economic model assessing biologic treatments versus nonbiologic therapy (NBT), to further illustrate the effect of using this evidence on cost-effectiveness estimates. The results from this illustrative example should be interpreted with caution and are not intended to be seen as an economic evaluation to inform current RA management. Table 1 summarizes the differences in the assumptions between the original economic model applied by Stevenson et al.^9^ and our updated model.

**Table 1:** Differences in the Assumptions between the Original Economic Model Applied by Stevenson et al.^9^ and the Updated Model Applied in Our Analysis Based on RWD

Assumption	Original Model	Updated Model
Effectiveness of second-line RTX	Equal to the effectiveness of first-line ABT	Estimated by applying target trial emulation using RWD from the BSRBR-RA register
Relationship between EULAR response categories and ACR response criteria	Estimated based on data from the US VARA register	Not required as RWD on EULAR response available from the BSRBR-RA directly

ABT, abatacept; ACR, American College of Rheumatology; BSRBR-RA, British Society of Rheumatology Biologics Register for Rheumatoid Arthritis; EULAR, European Alliance of Associations for Rheumatology; RTX, rituximab; RWD, real-world data; VARA, Veterans Affairs Rheumatoid Arthritis.

The remainder of this article is organized as follows. In the next section, we provide a detailed description of the target trial protocol that guided the RWD analysis and outline the economic model. The “Results” section presents the results from the TTE, including treatment effect estimates and the resulting cost-effectiveness estimates. We conclude the article with a discussion of our analysis and its implications on performing TTE using RWD for HTA in the fourth section.

## Methods

### TTE Using Register Data

The BSRBR-RA records RWD from routine clinical practice on individuals who have been prescribed biologic treatment for RA across the United Kingdom.^11^ The study cohort is a representative sample of the moderate-to-severe RA patient population in the United Kingdom. Data have been continually recorded since the register was established in 2001 and were available until 2015 for our analysis. Following the TTE method, we define below a protocol for a pragmatic randomized trial assessing the effectiveness of RTX versus NBT after failure with a first line of biologic treatment. We perform our analysis of the register data according to this protocol to minimize the influence of biases on the causal effect estimates.

#### Eligibility criteria

Individuals who were eligible for the target trial had been diagnosed with RA by the time of their enrollment into the register study. They had to have had active disease, defined by a disease activity score (DAS) 28 joint count measurement greater than 3.2 at the time of their target trial baseline date (see the “Follow-up Period” section for how the baseline was defined for each individual) so that the sample was representative of patients who may be moved on to a second-line biologic in clinical practice.^12^ We restricted the scope of the analysis to individuals who had stopped taking 1 of the following biologics (due to any reason, including inadequate response or intolerance) to be consistent with NICE clinical guidelines: ABT, adalimumab (ADA), certolizumab pegol (CTZ), etanercept (ETN), golimumab (GOL), infliximab (IFX), and TCZ. In addition, individuals in the register who were not taking methotrexate (MTX) at the time of their target trial baseline date were ineligible. This was to ensure that the causal effectiveness estimates would be generalizable to most patients who continue taking MTX with biologic treatment in clinical practice. We did not exclude individuals with a history of rheumatic autoimmune disease other than RA, or systemic involvement secondary to RA, as data on these characteristics were not readily available from the register. It was also not possible to emulate treatment discontinuation before randomization as most patients do not undergo a discontinuation period before switching to another biologic in practice.

#### Treatment strategies

We focused our analysis on individuals prescribed 1 of the following treatment strategies after failure with biologic treatment: 1) start taking RTX (in addition to MTX) (*n* = 1,360) or 3) NBT (including MTX) for at least 20 wk (*n* = 2,544). These were the treatment strategies that needed to be compared to inform the economic model implemented by Stevenson et al.^9^ An individual was considered to be taking RTX once they had started their first course of treatment (i.e., a course of RTX consists of 2 infusions separated by 2 wk) so that their defined baseline corresponded to their treatment initiation. We assumed that no changes were made to MTX dose or concurrent treatments (e.g., glucocorticoids, nonsteroidal anti-inflammatory drugs, DMARDs) because it was difficult to determine these characteristics at the time of a participant’s baseline from the data recorded in the register.

#### Assignment procedures

To emulate random treatment assignment, we adjusted for important differences in baseline characteristics between the treatment groups by applying IPTW based on propensity scores. This is the recommended approach in causal analysis of nonrandomized data to address confounders that do not change over time.^13^ We applied a logistic regression model to estimate a propensity score (i.e., the conditional probability of being assigned to the RTX treatment group) for each individual based on the following baseline characteristics: sex, disease duration, swollen and tender 28 joint counts, erythrocyte sedimentation rate, patient pain score, DAS28, Health Assessment Questionnaire (HAQ), number of previous DMARDs, previous biologic (ABT, ADA, ETN, IFX, TCZ), number of previous biologics, previous biologic discontinuation due to inefficacy, concurrent corticosteroid use, and weekly MTX dose. The characteristics were identified as important prognostic factors after discussion with a clinical expert. We accounted for missing values in baseline characteristics by multiple imputation using the predictive mean matching method^14^ to make the most efficient use of the data available.

#### Follow-up period

For each individual, the target trial baseline was defined as the date at which they started their assigned treatment strategy (i.e., the date of treatment initiation recorded in the register data). For individuals assigned to start taking RTX as second-line biologic treatment, this was the date at which they started their first course of RTX following failure with a first-line biologic treatment. For individuals assigned to NBT, this was the date at which they stopped taking their first-line biologic treatment. The follow-up date was defined as 24 wk after the baseline date, based on length of time used to assess treatment response in clinical practice. In the BSRBR-RA study, data are recorded at periodic follow-up visits (approximately every 6 mo), which may not coincide with the dates of treatment changes. In these cases, data were extracted corresponding to the closest follow-up visits to the baseline and follow-up dates as a compromise. These data were used to estimate the change in the outcome over the follow-up period representing the treatment response.

#### Outcome and causal contrast

We restricted the analysis to individuals who adhered to their assigned treatment strategy during the follow-up period to ensure that any difference in response was due to the treatment. For each individual, we calculated the change in the DAS28 outcome based on measurements at their baseline and follow-up dates. The baseline DAS28, and the change in DAS28 over the follow-up period, were used to classify each individual as a non-, moderate, or good responder to treatment according to the EULAR response criteria.^15^ The EULAR response is used to make treatment decisions in clinical practice and was a key parameter in the economic model developed by Stevenson et al.^9^ The causal treatment effect was measured as an odds ratio (OR) comparing the treatment groups in terms of the odds of achieving a better EULAR response after adjusting for differences in baseline characteristics to mitigate confounding. Modeling the EULAR response as an ordinal outcome (rather than a set of binary outcomes representing a response within each category) makes more efficient use of the data and is consistent with the approach used by Stevenson et al.^9^ to inform their economic model. The causal effect was estimated by applying an ordinal logistic regression model to predict EULAR response based on treatment group, after weighting each individual according to their IPTW to adjust for confounders.

### Meta-analysis

In addition to RWD from the BSRBR-RA study, summary trial-level data were extracted from a double-blind phase III RCT (REFLEX), which aimed to determine the efficacy of RTX in individuals with active RA who had failed to respond to at least 1 previous anti-TNF treatment.^16^ In the REFLEX trial, participants were randomized to receive RTX (*n* = 308) or placebo (*n* = 209), in addition to MTX. We chose to incorporate these data into the analysis because this was a pivotal trial on RTX and as such reported information highly relevant to its effectiveness. We performed a meta-analysis to combine the RWD and RCT data to estimate an overall treatment effect. We applied a conditional binomial fixed-effect meta-analysis model^2^ to estimate the absolute probability of each EULAR response category (none, moderate, good) associated with each treatment group (RTX versus NBT). The model was applied under a Bayesian framework using Markov chain Monte Carlo sampling.^17,18^

### Cost-Effectiveness Analysis

**Figure 1:**
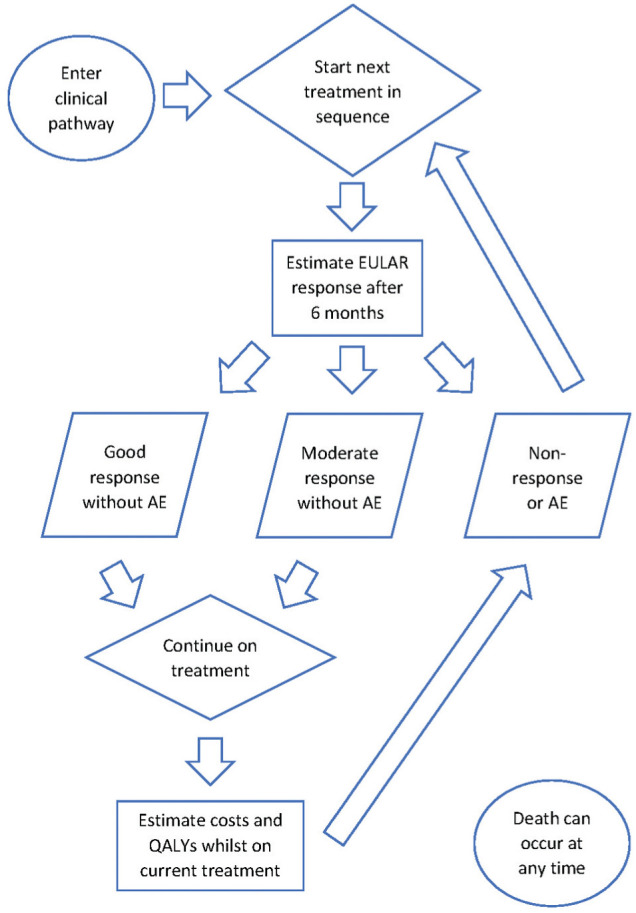
Flow diagram outlining the structure of the economic model developed by Stevenson et al.^9^ to assess the cost-effectiveness of clinical pathways associated with different biologic treatments and nonbiologic therapy for rheumatoid arthritis. This model employs discrete event simulation to estimate the lifetime costs and quality-adjusted life-years (QALYs) for each individual. AE, adverse event; EULAR, European Alliance of Associations for Rheumatology.

We applied the economic model developed by Stevenson et al.^9^ to estimate the cost-effectiveness of biologic treatments versus NBT. Figure 1 displays a schematic diagram outlining the economic model. A detailed description of the model is provided in the HTA by Stevenson et al.^9^ (see pages 237–66). In the model, the EULAR response probability for each treatment determines the time spent by an individual on that treatment. The model estimates the lifetime costs and quality-adjusted life-years (QALYs) for the treatment strategies associated with 8 different biologics and with NBT. RTX is assumed to be the second line of treatment in each biologic strategy. Our analysis aimed to compare the cost-effectiveness of an average biologic strategy versus an NBT strategy. As such, we used the mean average lifetime costs and QALYs across biologic strategies to estimate an incremental cost-effectiveness ratio (ICER). We applied the model under 3 separate scenarios using a different evidence source to inform EULAR response probability estimates for RTX as model inputs each time: RCT data alone, RWD alone, and a meta-analysis of RCT data and RWD.

In the original model, the clinical effectiveness estimates for ABT as a first line of biologic treatment were assumed to be generalizable to ABT as a second-line biologic, and the effectiveness of RTX as a second-line biologic was assumed to be equivalent to ABT. The plausibility of these assumptions is questionable given that 1) a biologic is less effective when given as a second-line treatment^19^ and 2) RTX and ABT have a different mechanism of action. Thus, these assumptions have the potential to bias the cost-effectiveness results in favor of the biologic strategies when compared with the NBT strategy. We explored the influence of these assumptions by applying the economic model using the pooled EULAR response probability estimates for RTX based on a meta-analysis of RWD and RCT data. Aside from this difference in model inputs, our application of the model was unchanged from the original analysis by Stevenson et al.^9^ We performed a probability sensitivity analysis (PSA), with 600 iterations of 2,000 individuals, to incorporate uncertainty in model inputs into the cost-effectiveness results.

## Results

### TTE Using Register Data

We extracted RWD from the BSRBR-RA according to the target trial protocol described in the “Target Trial Emulation Using Register Data” section. Table 2 summarizes the baseline characteristics for the target trial participants by treatment group, based on the register data. A greater number of participants were assigned to the NBT strategy (*n* = 2,544) compared with the RTX strategy (*n* = 1,360). There was a larger percentage of females in the RTX group (83.2%) compared with the NBT group (78.2%). A typical participant in the RTX group had been diagnosed with RA for longer than a typical participant in the NBT group (median 16 v. 14 y). Participants in the RTX group had greater disease activity compared with the NBT group (median DAS28 6 v. 5). Those in the RTX group were more likely to have discontinued previous biologic therapy due to inefficacy compared with the NBT group (60.6% v. 44.5%). Table A1 in Appendix A presents a side-by-side comparison of the baseline characteristics for individuals included in the TTE analysis with participants in the RCT performed by Cohen et al.^16^

**Table 2:** Summary of Baseline Characteristics by Treatment Group for Individuals Eligible for the Target Trial^a^

Characteristic	Nonbiologic Therapy	Rituximab
*n*	2,544	1,360
Age, y	61 (52, 68)	60 (51, 67)
Female, No. (%)	1,989 (78.2)	1,132 (83.2)
Disease duration, y	14 (8, 22) (NA = 21)	16 (10, 23) (NA = 8)
Tender joint count (28 joints assessed)	8 (4, 16) (NA = 399)	10 (5, 17) (NA = 411)
Swollen joint count (28 joints assessed	5 (2, 10) (NA = 399)	6 (3, 10) (NA = 410)
Erythrocyte sedimentation rate, mm/h	33 (19, 54) (NA = 570)	35 (20, 59) (NA = 519)
C-reactive protein, mg/dL	1 (1, 4) (NA = 1,501)	2 (1, 4) (NA = 836)
Patient global assessment (0–100)	61 (44, 79) (NA = 431)	66 (49, 80) (NA = 434)
Disease Activity Score in 28 joints	5.4 (4.3, 6.4)	5.6 (4.6, 6.6)
Health Assessment Questionnaire score, mean (SD)	2.0 ± 0.6 (NA = 132)	1.9 ± 0.6 (NA = 44)
RF positive, No. (%)	1,660 (65.9) (NA = 25)	923 (68.2) (NA = 6)
Comorbidity, No. (%)	1,625 (64.9) (NA = 41)	850 (63.2) (NA = 16)
No. of previous cDMARDs	3 (2, 4)	3 (2, 4)
Previous biologic taken, No. (%)		
Abatacept	44 (1.7)	7 (0.5)
Adalimumab	1,071 (42.1)	779 (57.3)
Certolizumab	16 (0.6)	14 (1)
Etanercept	1,002 (39.4)	805 (59.2)
Golimumab	9 (0.4)	4 (0.3)
Infliximab	1,304 (51.3)	652 (47.9)
Tocilizumab	123 (4.8)	17 (1.2)
Inadequate efficacy of biologic, No. (%)	1,133 (44.5)	824 (60.6)
Use of glucocorticoids as baseline, No. (%)	710 (42.9) (NA = 890)	563 (50.6) (NA = 247)
Weekly dose of MTX at baseline, mg	10 (8, 13) (NA = 1,884)	8 (5, 10) (NA = 897)

cDMARDs, conventional disease-modifying antirheumatic drugs; MTX, methotrexate; NA, number of observations for which data were missing/not available; RF, rheumatoid factor.

a Statistics are presented as median (interquartile range), unless stated otherwise.

We applied an ordinal logistic regression model to compare the treatment groups in terms of the odds of achieving a better EULAR response and to predict the EULAR response based on treatment group with (and without) adjustment by IPTW. Table 3 presents the ORs and EULAR response probabilities estimated in this analysis. The unadjusted OR estimate indicates that participants taking RTX had 37% increased odds of achieving a better EULAR response compared with those taking NBT (OR = 1.37, 95% credible interval [CrI]: 1.18, 1.58). The effect was similar after applying IPTW to adjust for differences in baseline characteristics between the treatment groups, where the increase in odds was estimated to be 39% (OR = 1.39, 95% CrI: 1.25, 1.55).

**Table 3:** Odds Ratio (Mean and 95% Confidence Interval) and Response Probability Estimates, Comparing Rituximab versus Nonbiologic Therapy in Terms of the European Alliance of Associations for Rheumatology Outcome, with and without Adjustment by Inverse Probability of Treatment Weighting (IPTW)^a^

Outcome	Nonbiologic Therapy	Rituximab
Odds ratio		
Unadjusted	Reference	1.37 (1.18, 1.58)
Adjusted for IPTW	Reference	1.39 (1.25, 1.55)
Response probability		
Unadjusted		
At least moderate	0.179 (0.154, 0.204)	0.219 (0.207, 0.232)
Good	0.065 (0.057, 0.073)	0.086 (0.085, 0.087)
Adjusted for IPTW		
At least moderate	0.174 (0.145, 0.203)	0.215 (0.121, 0.309)
Good	0.066 (0.058, 0.076)	0.090 (0.065, 0.123)

a Estimates are based on the target trial emulation using real-world data from the British Society for Rheumatology Biologics Register for Rheumatoid Arthritis.

The model was also applied to estimate the probability of an at least moderate, and a good, EULAR response on each treatment. Participants taking RTX were more likely to achieve either a moderate or good response compared with those taking NBT (probability = 0.219 v. 0.179) and were also more likely to achieve a good response (probability = 0.086 v. 0.065). These estimates were similar after adjustment with IPTW.

### Meta-Analysis of RWD and RCT Data

In the original analysis by Stevenson et al.,^9^ the EULAR response probabilities associated with RTX were assumed to be 0.696 (95% CrI: 0.345, 0.907) and 0.250 (0.058, 0.559), for an at least moderate and a good response, respectively. In an RCT conducted by Cohen et al,^16^ among participants allocated to receive RTX, the proportion who achieved at least a moderate response was 0.650, and the proportion who achieved a good response was 0.150. The estimates from our analysis using RWD were more modest compared with the estimates based on the RCT data, reflecting the gap between the efficacy and effectiveness of RTX. We performed a meta-analysis combining the RWD and the RCT data that estimated the pooled EULAR response probabilities to be 0.336 (0.119, 0.636) and 0.096 (0.019, 0.297), for an at least moderate and a good response, respectively. The pooled estimates were entered into the economic model to perform the cost-effectiveness analysis.

We also performed a regression analysis to statistically compare the difference in EULAR response probability estimates from the RCT and RWD sources. The RWD population had significantly lower odds of achieving a EULAR response compared with the RCT population: OR 0.30 (95% confidence interval: 0.23, 0.37). This suggests that the estimates assumed by Stevenson et al.^9^ may not be representative of the effectiveness of RTX in UK clinical practice. Indeed, patients in the RWD population are older, have a longer disease duration, have lower disease activity at baseline, and are less likely to be RF positive than patients in the RCT population.

### Cost-Effectiveness Analysis

Table 5 presents the cost-effectiveness results (as mean and 95% CrI estimates) from a PSA comparing an average biologic treatment strategy with an NBT strategy, in terms of the lifetime QALYs, lifetime costs, and ICERs. The results corresponding to the analysis based on the RTX effect estimates from the REFLEX RCT performed by Cohen et al.^16^ are listed under the column titled “RCT Data.” The results based on our TTE analysis using RWD from the BSRBR-RA are listed under the column titled “RWD.” The results based on pooling the effect estimates from the RCT data and RWD are listed under the column titled “RCT Data + RWD.”

In the RCT data analysis, the biologic strategy was associated with a larger treatment benefit compared with the NBT strategy (mean QALYs: 5.60 v. 4.79). In the RWD analysis, the size of the difference in treatment benefit was smaller relative to the RCT data analysis because there was a decrease in the QALYs associated with the biologic strategy (mean QALYs: 5.27 v. 4.81). This is expected as the RTX effect was more modest when estimated from RWD compared with RCT data, which would decrease the probability of treatment response, and hence QALYs, associated with taking RTX when assessing the biologic strategy in the economic model. When combining the RWD and RCT data, the benefit of the biologic strategy was also larger than that of the NBT (mean QALYs: 5.35 v. 4.84), as expected. The QALY estimate for RTX was smaller than the estimate from the analysis of RCT data alone but larger than the estimate obtained from RWD.

In the RCT data analysis, the lifetime costs associated with the biologic strategy were much greater than the costs associated with the NBT strategy (mean costs: £70,000 v. £42,200). The size of this difference in costs decreased in the RWD analysis, due to a decrease in the costs associated with the biologic strategy (mean costs: £63,300 v. £42,300 for NBT). According to the description of the economic model (see pages 237–42 in Stevenson et al.^9^), the smaller RTX effect estimates obtained from the RWD analysis would have led to a higher probability of no response to treatment (in terms of EULAR criteria) used as an input to the economic model (see Table 4 for the RTX response probabilities used as model inputs). As such, patients would be modeled to stop RTX treatment and move to the next treatment in the sequence more quickly. This shorter time on treatment would mean lower treatment and administration costs associated with RTX and hence lower overall costs associated with the biologic strategy. In the RCT data + RWD analysis, the difference in cost estimates was similar to that from the RWD-only analysis (mean costs: £64,700 v. £42,700 for RTX and NBT, respectively).

**Table 4:** Pooled EULAR Response Probability Estimates for Second-Line Rituximab, from a Meta-Analysis of Observed Estimates from the “REFLEX” RCT by Cohen et al.^16^ and Estimates Obtained from the TTE using Real-World Data from the BSRBR-RA

		EULAR Response Probability	
Evidence Source	*n*	At Least Moderate	Good
REFLEX RCT	298	0.650	0.150
BSRBR-RA (TTE)	1,360	0.215	0.090
Pooled	1,658	0.336 (95% CrI: 0.119, 0.636)	0.096 (0.019, 0.297)

BSRBR-RA, British Society for Rheumatology Biologics Register for Rheumatoid Arthritis; CrI, credible interval; EULAR, European Alliance of Associations for Rheumatology; RCT, randomized controlled trial; TTE, target trial emulation.

**Table 5:** Mean and 95% Credible Interval Estimates for Lifetime QALYs, Lifetime Costs (£), and ICERs (£ per QALY Gained) Comparing an Average Biologic Strategy versus an NBT Strategy^a^

Outcome	Strategy	RCT Data	RWD	RCT Data + RWD
QALYs				
	NBT	4.79 (3.63, 5.81)	4.81 (3.72, 5.85)	4.84 (3.78, 5.81)
	Biologic	5.60 (4.16, 6.84)	5.27 (4.06, 6.39)	5.35 (4.14, 6.60)
Costs				
	NBT	42,200 (27,800, 55,000)	42,300 (29,000, 54,900)	42,700 (30,000, 55,000)
	Biologic	70,000 (51,500, 86,400)	63,500 (48,300, 77,500)	64,700 (50,300, 80,700)
ICER				
	Biologic v. NBT	34,700 (29,500, 44,000)	46,800 (37,600, 58,900)	46,300 (29,600, 72,300)

ICER, incremental cost-effectiveness ratio; NBT, nonbiologic therapy; QALY, quality-adjusted life-year; RCT, randomized controlled trial; RWD, real-world data.

Results correspond to analyses based on the rituximab effect estimates originally assumed by Stevenson et al.^9^ (RCT data), the estimates obtained from the target trial emulation using RWD, and the estimates obtained from pooling RCT data and RWD (RCT data + RWD). Results were obtained from a probability sensitivity analysis based on the full probability distribution for the effect estimates. Costs and ICERs have been rounded to the nearest £100. The mean (and credible interval) ICER estimates summarize the distribution of ICERs calculated within each iteration of the probability sensitivity analysis.

The ICER comparing the biologic strategy with the NBT strategy was larger when estimated based on RWD compared with the RCT data (mean ICER: £46,800 v. £34,700 per QALY gained). The estimate from the RCT data + RWD analysis was similar to that from the RWD-only analysis (mean ICER: £46,300 v. £46,800 per QALY gained).

**Figure 2:**
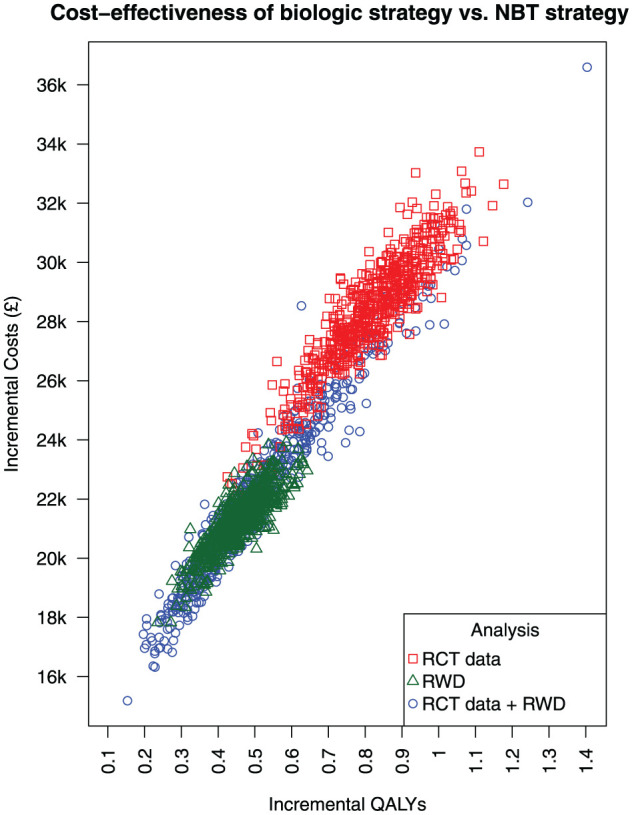
Cost-effectiveness plane illustrating incremental quality-adjusted life-years (QALYs) gained against incremental costs (£) comparing an average biologic treatment strategy versus a nonbiologic therapy (NBT) strategy. Results correspond to analyses based on the rituximab effect estimates from the REFLEX RCT by Cohen et al.^16^ (RCT data) from our target trial emulation using real-world data (RWD) and from pooling both sets of estimates (RCT data + RWD). Each point represents 1 iteration from the probability sensitivity analysis.

Figure 2 illustrates the incremental QALYs gained plotted against incremental costs (£) comparing the biologic strategy versus the NBT strategy for the 3 separate analyses: RCT data, RWD, and RCT data + RWD. Each point represents the estimate from 1 PSA iteration.

In the RCT data analysis (red points), the biologic strategy was associated with greater QALYs gained compared with the NBT strategy, with the difference in QALYs gained ranging from approximately 0.5 to 1.1. This association was also evident in the RWD analysis (green points), although the QALYs gained were lower, ranging from approximately 0.3 to 0.7. In the RCT data + RWD analysis (blue points), the QALYs had a broader spread ranging from 0.2 to 1.4.

In the RCT data analysis, the biologic strategy was associated with greater costs compared with the NBT strategy, with the incremental costs ranging from approximately £24,000 to £34,000. In the RWD analysis, this difference in costs was lower, ranging from approximately £18,000 to £24,000. The costs also had a broader spread in the RCT data + RWD analysis, ranging from approximately £16,000 to £36,000. For all analyses, there were no PSA iterations in which the biologic strategy was estimated to have lower costs, or lower QALYs gained, compared with the NBT strategy.

**Figure 3:**
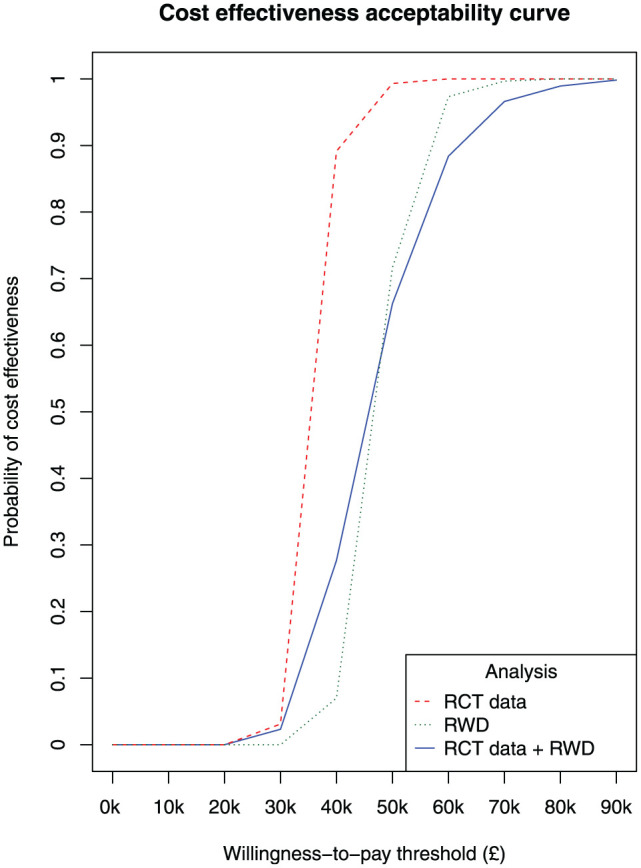
Cost-effectiveness acceptability curve illustrating the probability of cost-effectiveness at different willingness-to-pay thresholds (£) comparing an average biologic treatment strategy versus a nonbiologic therapy (NBT) strategy. Results correspond to analyses based on the rituximab effect estimates from the REFLEX RCT by Cohen et al.^16^ (RCT data), from our target trial emulation using real-world data (RWD), and from pooling both sets of estimates (RCT data + RWD). Each point represents 1 iteration from the probability sensitivity analysis.

Figure 3 shows the probability of cost-effectiveness at different willingness-to-pay thresholds (£) comparing an average biologic treatment strategy versus an NBT strategy, for the 3 separate analyses: RCT data, RWD, and RCT data + RWD. All analyses show the biologic strategy as highly unlikely to be cost effective up until approximately £30,000 per QALY. There is a steep increase in the probability of cost-effectiveness between the £30,000 and £60,000 thresholds, and the increase is steeper when the probability estimates are based on the RCT data compared with RWD. The RCT data indicate that the biologic strategy has a 50/50 chance of being cost-effective at the £40,000 threshold, whereas the RWD indicate that this chance occurs at the £50,000 threshold. The biologic strategy is highly likely to be cost-effective above £50,000 per QALY gained based on the RCT data, but when based on RWD, this conclusion can be made only above approximately £70,000.

## Discussion

### Summary

In this article, we illustrated the TTE method using RWD from the BSRBR-RA to estimate the effectiveness of RTX as a second line of biologic treatment for RA in terms of the EULAR response categories. We estimated a more modest effect compared with previous analyses based on RCT evidence alone. This discrepancy is most likely due to differences in characteristics, between the broader population represented by the BSRBR-RA cohort and the narrower population eligible for a clinical trial, which influence treatment response. We also illustrated the knock-on impact on cost-effectiveness estimates by entering the effect estimates from our TTE analysis into an economic model assessing biologic treatments versus NBT. We found that the ICER comparing biologic and NBT treatment strategies was higher when the RTX effect estimates were based on RWD compared with RCT data.

### Strengths and Limitations

A strength of our use of RWD was that we were able to estimate a treatment effect in terms of the EULAR response outcome directly, which is used in the economic model to calculate cost-effectiveness estimates. RCTs assessing biologic treatments for RA commonly report data on effectiveness in terms of the American College of Rheumatology response criteria, which require mapping to the EULAR response. However, the BSRBR-RA study collects data on the DAS28 outcome, which is the basis for the EULAR response categories.

There were limitations with applying the TTE method using RWD in our case study. We defined a target trial protocol for a pragmatic RCT in which participants were aware of their assigned treatment. Therefore, we could not account for a placebo effect in our analysis, and this is likely to have led to an overestimate of the treatment effect because individuals are prone to score better on patient-reported outcomes (e.g., pain) when aware of their treatment.^20^

Ideally, the treatment effect would be estimated based on the change in disease activity measured from baseline (i.e., immediately before treatment initiation) to the end of the follow-up period such as in a clinical trial. In the BSRBR-RA, data on disease activity were recorded at periodic follow-up hospital appointments approximately every 6 mo. The dates at which disease activity was measured (in terms of the DAS28 outcome) did not always coincide with the biologic treatment changes that were used to define the baseline and follow-up dates for each target trial participant. We attempted to mitigate this by extracting RWD from the closest follow-up visits to the baseline and follow-up dates. The register was not originally intended to provide evidence on treatment effectiveness, so the DAS28 measured at the time of treatment decisions is not always recorded, although this may not be an issue with other RWD sources. This could introduce bias in the treatment effect estimate, particularly in cases in which there is a large time difference between the DAS28 measurement and the treatment decision. For example, where the DAS28 is measured after the start of the treatment, it may not truly represent the initial reduction in disease activity due to the treatment response. Thus, the change in disease activity from baseline to follow-up will be underestimated, which will bias the treatment effect toward lower values.

The EULAR response probabilities associated with second-line RTX estimated in our TTE analysis were much lower than those based on RCT data (see Table 4). In the tightly controlled clinical trial setting, patients are more likely to experience disease remission and greater decreases in the DAS28 and HAQ outcome measures. Our study was focused on illustrating the applicability of the TTE method using RWD. Although we did not explore the reasons for the discrepancy, future work could investigate how differences in the RCT and RWD populations/settings influence the differences in effect estimates obtained from each evidence source. It would also be useful to benchmark a TTE analysis by emulating an existing RCT, based on population and study design, and comparing the effect estimates obtained from each. Previous studies assessing anti-TNF drugs have found a lower treatment response when estimated from RWD sources compared with RCTs.^21,22^

In our analysis, we did not adjust for participants assigned to both treatment groups. For example, a participant could stop taking a first line of biologic treatment and take NBT for a period of time (treatment group 1), before starting RTX as a second line of biologic treatment (treatment group 2). There may be differences in the characteristics between these participants and those who immediately started RTX as a second line of biologic treatment, which could introduce heterogeneity in the treatment effect estimate.

We assessed the cost-effectiveness of an average biologic treatment strategy versus NBT by averaging over the lifetime costs and QALYs estimates from 8 separate biologic treatment strategies included in the economic model developed in Stevenson et al.^9^ (see Table 139 on page 239 in Stevenson et al.). We did not consider differences in cost-effectiveness between biologic treatments, which may be likely given that some of the included treatments belong to different therapeutic classes.

One of the benefits of RWD from longitudinal cohort studies, such as the BSRBR-RA register, is the possibility of modeling the effectiveness of treatment sequences. We focused our TTE analysis on estimating the effectiveness of RTX given as a second-line biologic to provide supplemental evidence where this was lacking from randomized trials. Data from the BSRBR-RA register have been used to assess the effectiveness of sequences of biologic treatments,^19^ and future work could look at using this evidence to inform an economic model.

We applied IPTW to adjust for differences in the measured baseline characteristics between the treatment groups. This was intended to mitigate bias in the treatment effect estimate due to differences in confounders—characteristics that are associated with both treatment allocation and the outcome. However, even after applying such adjustment techniques, effect estimates based on data from nonrandomized sources can still be subject to (unquantifiable) bias due to unmeasured confounders. We did not grade the quality of evidence from the register and assumed that the aforementioned techniques were sufficient to mitigate confounding.

In our TTE analysis, individuals assigned to the RTX treatment group were more likely to have stopped previous biologic therapy due to inefficacy compared with individuals assigned to NBT (60.6% v. 44.5%). This may be because the NBT group contained a higher proportion of individuals who cannot take a biologic treatment due to contraindication with other medications. Although we adjusted for the difference in the proportion of individuals who stopped previous biologic therapy due to inefficacy between the 2 treatment groups, this may not have completely mitigated this selection bias.

In the cost-effectiveness analysis, the number of PSA iterations was limited to 600 due to time constraints. Performing this analysis with a larger number of iterations would provide greater confidence in the stability of the mean ICER estimates.

## Conclusion

Our findings suggest that RWD can provide supplemental evidence on the effectiveness of a treatment in clinical practice, which can make a notable difference to the cost-effectiveness estimates informing HTA decision making. Future work should explore the application of TTE using RWD sources to estimate treatment effectiveness in other case studies. Further development of evidence synthesis methods for combining RWD and RCT data on treatment effectiveness, while accounting for the strengths and limitations of each evidence source, would also be useful.

Our study aimed to illustrate how the TTE method can be applied to RWD to provide supplemental evidence on treatment effectiveness. This illustrative example supports the potential usefulness of this approach in HTA to aid policy makers in decision problems in which randomized evidence on clinical effectiveness is limited and/or from a narrowly defined population that is not representative of the wider population considered in the decision problem.

The HTA report assessing biologics for RA, which we used as a case study, was published in 2016.^9^ Since then, the management of RA has changed due to the availability of new RCT data on comparators (e.g., Janus kinase inhibitors) and on treatment sequences not including RTX. Consequently, our analysis should not be seen as an economic evaluation to inform current RA management but instead as an illustrative example of the TTE method in HTA.

## Appendix

**Table A1:** Summary of Baseline Characteristics by Treatment Group for Individuals Eligible for the Target Trial in the British Society for Rheumatology Biologics Register for Rheumatoid Arthritis (BSRBR-RA) and the Randomized Controlled Trial (RCT) performed by Cohen et al.^16,a^

Characteristic	Nonbiologic Therapy (BSRBR-RA)	Rituximab (BSRBR-RA)	Placebo (RCT)	Rituximab (RCT)
*n*	2,544	1,360	209	308
Age, y	59.4 ± 12.4	58.8 ± 11.9	52.8 ± 12.6	52.2 ± 12.2
Female, No. (%)	1,989 (78.2)	1,132 (83.2)	169 (81)	251 (81)
Disease duration, y	16.1 ± 10.3	17.4 ± 9.9	11.7 ± 7.7	12.1 ± 8.3
Erythrocyte sedimentation rate, mm/h	39.5 ± 27.2	42.6 ± 28.6	48.4 ± 27.8	48.0 ± 25.5
C-reactive protein, mg/dL	3.0 ± 3.8	3.0 ± 3.4	3.8 ± 4.1	3.7 ± 3.8
Disease Activity Score in 28 joints	5.4 ± 1.3	5.6 ± 1.3	6.8 ± 1.0	6.9 ± 1.0
Health Assessment Questionnaire score	2.0 ± 0.6	1.9 ± 0.6	1.9 ± 0.5	1.9 ± 0.6
RF positive, No. (%)	1,660 (65.9)	923 (68.2)	165 (79)	242 (79)
No. of previous DMARDs	3.1 ± 1.7	3.2 ± 1.7	2.4 ± 1.8	2.6 ± 1.8
Infliximab	1,304 (51.3)	652 (47.9)	169 (81)	219 (71)
Adalimumab	1,071 (42.1)	779 (57.3)	38 (18)	71 (23)
Etanercept	1,002 (39.4)	805 (59.2)	104 (50)	168 (55)
No. of previous anti-TNFs	1.3 ± 0.6	1.7 ± 0.7	1.5 ± 0.67	1.5 ± 0.67
1 anti-TNF, No. (%)	1,689 (66.4)	591 (43.5)	125 (60)	186 (60)
2 anti-TNFs, No. (%)	664 (26.1)	612 (45)	65 (31)	94 (31)
3 anti-TNFs, No. (%)	119 (4.7)	141 (10.4)	19 (9)	28 (9)
Inadequate efficacy of biologics, No. (%)	1,133 (44.5)	824 (60.6)	188 (90)	283 (92)
Use of glucocorticoids as baseline, No. (%)	710 (42.9)	563 (50.6)	127 (61)	200 (65)
Weekly dose of MTX at baseline, mg	11.1 ± 12.0	9.8 ± 12.4	16.7 ± 9.9	16.4 ± 8.8

DMARDs, disease-modifying anti-rheumatic drugs; MTX, methotrexate; RF, rheumatoid factor; TNF, tumor necrosis factor.

a Statistics are presented as mean ± standard deviation, unless stated otherwise. The number of observations for which data were missing/NA (not available) are given in brackets.
